# Renal Protective Effects of 17β-Estradiol on Mice with Acute Aristolochic Acid Nephropathy

**DOI:** 10.3390/molecules21101391

**Published:** 2016-10-18

**Authors:** Min Shi, Liang Ma, Li Zhou, Ping Fu

**Affiliations:** 1 Division of Nephrology, Department of Internal Medicine, West China Hospital of Sichuan University, Chengdu 610041, Sichuan, China; minshi0616@163.com (M.S.); Liang_m@scu.edu.cn (L.M.); 2 Kidney Research Institute, West China Hospital of Sichuan University, Chengdu 610041, Sichuan, China

**Keywords:** estradiol, aristolochic acid nephropathy, apoptosis, renal tubular epithelial cell, p53

## Abstract

Aristolochic acid nephropathy (AAN) is a progressive kidney disease caused by a Chinese herb containing aristolochic acid. Excessive death of renal tubular epithelial cells (RTECs) characterized the acute phase of AAN. Therapies for acute AAN were limited, such as steroids and angiotensin-receptor blockers (ARBs)/angiotensin-converting enzyme inhibitors (ACEIs). It was interesting that, in acute AAN, female patients showed relative slower progression to renal failure than males. In a previous study, female hormone 17β-estradiol (E2) was found to attenuate renal ischemia-reperfusion injury. Thus, the aim of this study was to investigate the potential protective role of E2 in acute AAN. Compared with male C57BL/6 mice of acute AAN, lower serum creatinine (SCr) and less renal injury, together with RTEC apoptosis in females, were found. Treatment with E2 in male AAN mice reduced SCr levels and attenuated renal tubular injury and RTEC apoptosis. In the mice kidney tissue and human renal proximal tubule cells (HK-2 cells), E2 both attenuated AA-induced cell apoptosis and downregulated the expression of phosphor-p53 (Ser15), p53, and cleaved-caspase-3. This study highlights that E2 exhibited protective effects on the renal injury of acute AAN in male mice by reducing RTEC apoptosis, which might be related to inhibiting the p53 signaling pathway.

## 1. Introduction

**Figure 1:**
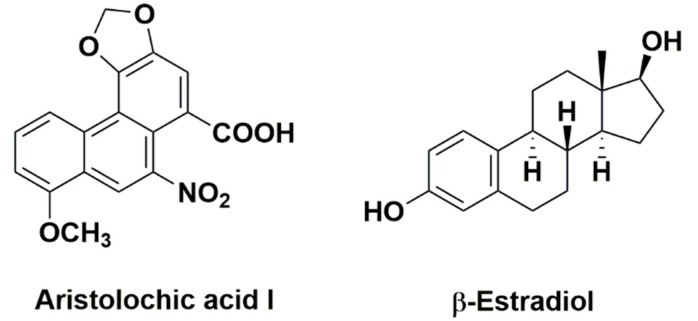
The chemical structure of aristolochic acid I and estradiol.

Aristolochic acid nephropathy (AAN), initially called Chinese herb nephropathy, was firstly reported in Belgium in a group of female patients who followed a slimming regimen containing Chinese herbs [1,2]. The causative agent of AAN is aristolochic acid (AA) [3]. Aristolochic acid was found in *Aristolochia* and *Asarum *species including more than 10 derivatives, the most prevalent of which were aristolochic acid I (Figure 1) and II, both of which have potentially nephrotoxic and carcinogenic effects [3,4]. Patients with AAN showed a rapidly progressive interstitial nephritis that led to end stage renal diseases (ESRDs) and urothelial malignancy [1,5,6,7]. It has been demonstrated that RTECs are the main target of AA [4,8]. Although the AA-induced RTEC injury was not completely clear, it was thought that cell apoptosis played key roles in acute AAN leading to irreversible proximal tubular atrophy [4,8,9]. Therapies for acute AAN were limited, such as steroids and angiotensin-receptor blockers (ARBs)/angiotensin-converting enzyme inhibitors (ACEIs).

Women show slower progression of renal injury compared with men in chronic renal disease [10,11]. Male are more likely to experience acute kidney injury (AKI) than females [12,13]. Physicians in Belgium defined AAN via a case series from a group of females with a slimming regimen [1]. However, in some other studies, involving male and female patients both, it was found that, in acute AAN, female patients show relative slower progression to renal failure than do males [14,15]. In our observation, in acute AAN cases (unpublished data), 4 in 5 patients were male and needed prompt hemodialysis. Female patients suffering from chronic AAN received maintenance hemodialysis a year later.

In fact, it has been demonstrated that 17β-estradiol (E2, Figure 1) has a renal-protective effect on renal injury in animal models of renal ischemia-reperfusion (RIR) and diabetic nephropathy [11,16,17,18,19,20]. The renoprotective role of E2 in RIR may be that E2 can suppress renal sympathetic nerve activity enhanced during renal ischemia [17]. Furthermore, the renoprotective effect of E2 in RIR is due to activation of the PI3K/Akt pathway followed by increased phosphorylation of the endothelial nitric oxide synthase (eNOS) [20]. In progression of renal damage, estrogen upregulates the renal synthesis of nitric oxide and influences the synthesis and release of various growth factors, hormones, and cytokines [10]. It has been shown that E2 influences mesangial cell biology to inhibit kidney disease progression [10,21]. E2 suppresses the synthesis of collagen and the apoptosis of mesangial cells induced by transforming growth factor β1 (TGF-β1) [10]. It has also been demonstrated that E2 protects podocytes from apoptosis induced by TGF-β1 and tumor necrosis factors-α (TNF-α) in animal models [22]. E2 also prevents testosterone-induced apoptotic damage in renal tubular epithelial cells (RTECs) [23]. Therefore, E2 might be a potential therapy for acute AAN.

In our previous study, we found that AA induced RTEC apoptosis via posttranslational activation of p53 to promote renal injury in acute AAN [8]. Thus, the blockage of RTEC apoptosis was favored for treating the acute AAN. Therefore, in this study, in vivo and in vitro experiments were set up to test our hypothesis about the potential protective role of female hormone 17β-estradiol in acute AAN and to explore the underlying mechanism related to p53.

## 2. Results

### 2.1. AA-Induced Acute AAN Was More Severe in Male than in Female Mice

**Figure 2:**
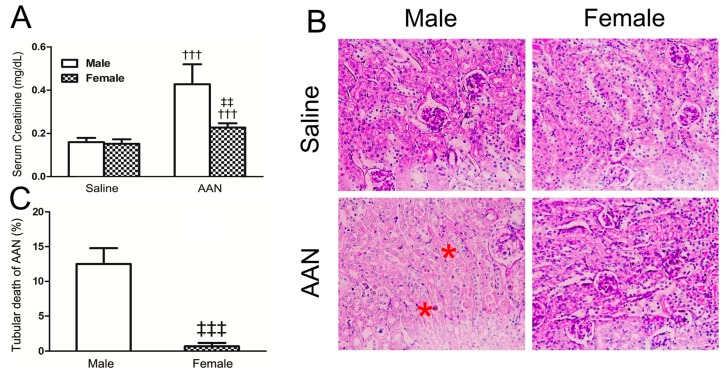
AA-induced acute AAN in mice at day 5. (**A**) Serum creatinine at day 5 after induction of acute AAN; (**B**) Histology (PAS-stained sections) at day 5 after induction of acute AAN. Note that there were many tubules with typical coagulative necrosis morphology (asterisk) in male mice with acute AAN; (**C**) Quantitative analysis of tubular death. Data were expressed as means ± SD for groups of 6 mice. ^†††^ *p* < 0.001 vs. saline control mice; ^‡‡^ *p* < 0.01, ^‡‡‡^ *p* < 0.001 vs. male mice with acute AAN. Magnification, ×200.

Compared with respective control mice, the SCr value of male in the AAN group was markedly elevated and that of the female was slight increased with statistical significance (male: 0.43 ± 0.09 mg/dL vs. 0.16 ± 0.02 mg/dL, *p* < 0.001; female: 0.23 ± 0.02 mg/dL vs. 0.15 ± 0.02 mg/dL, *p* < 0.001) (Figure 2A). The SCr levels in the female AAN group were remarkably lower than that in the male AAN group (*p* < 0.01) (Figure 2A). The PAS staining of kidney tissue exhibited a typical pathologic feature of male AAN mice, which characterized that the bare basement membrane due to RTECs detached from the renal tubular basement (Figure 2B). Furthermore, mild renal tubule injuries, characterized by dilated tubules with an absence of the bare basement membrane, were observed in the female AAN group. The ratio of AA-induced injured cortical tubule, also indicated by apoptotic and necrotic RTECs and dilated tubules, was up to 10%–15% in male mice (Figure 2C), whereas the proportion of tubular death of females was fairly small in contrast to male AAN mice (*p* < 0.001).

**Figure 3:**
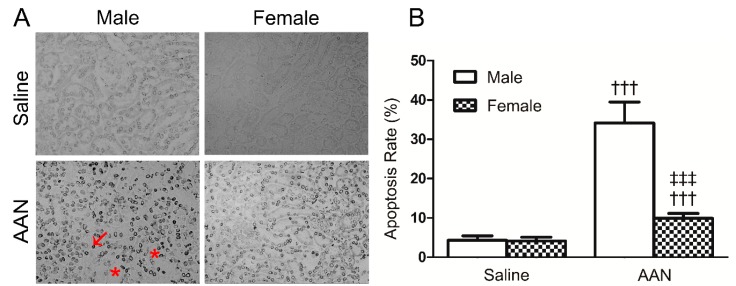
AA-induced RTEC apoptosis in mice at day 5. (**A**) RTEC apoptosis in acute AAN mice as detected by TUNEL assay. Note that there were many TUNEL^+^ apoptotic cells (arrow) in the area with cortical tubular necrosis (asterisk) in a male mouse with acute AAN; (**B**) Quantitative analysis of TUNEL^+^ apoptotic cells. Data were expressed as means ± SD for groups of 6 mice. ^†††^ *p* < 0.001 vs. saline control mice; ^‡‡‡^ *p* < 0.001 vs. male mice with acute AAN. Magnification, ×200.

As shown in Figure 3, the number of TUNEL^+^ apoptotic cells was significantly increased in the male AAN mice (34.14% ± 5.35%); however, the apoptosis rate of the RTECs in the female AAN group was slightly low (9.91% ± 1.34%). The results suggest that female mice are resistant to AA toxicity with the low RTEC apoptosis in comparison with male AAN mice.

### 2.2. E2 Had a Protective Effect on Renal Injury in Male AAN Mice

**Figure 4:**
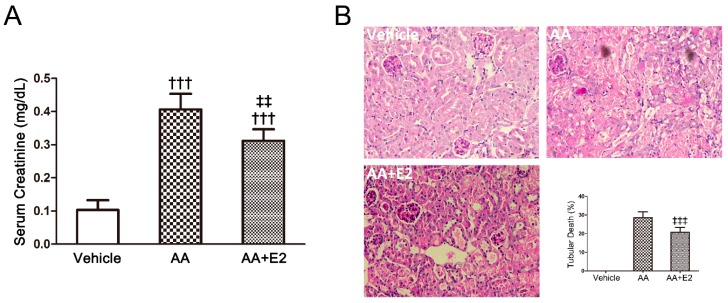
AA-induced acute AAN in male mice at day 5 were attenuated by treatment with E2. (**A**) Measurement of serum creatinine; (**B**) Histology (PAS-stained sections) and quantitative analysis of tubular death. Note that there were less necrotic tubules in AA + E2 group compared with AA group. Data were expressed as means ± SD for groups of 6 mice. ^†††^ *p* < 0.001 vs. vehicle group; ^‡‡^ *p* < 0.01, ^‡‡‡^ *p* < 0.001 vs. AA group. Magnification, ×200.

We established the AAN model in male mice to test whether E2 attenuated renal injury in acute AAN. Pretreatment with E2 (2 mg/kg/day) in male mice of acute AAN was found to reduce SCr levels (0.31 ± 0.03 mg/dL vs. 0.41 ± 0.05 mg/dL, *p* < 0.01) and alleviate tubular injury by PAS staining of renal tissues (Figure 4). The proportion of dead tubules was reduced from 28.6% ± 3.1% to 20.7% ± 2.7% by the E2 treatment (*p* < 0.001). 

**Figure 5:**
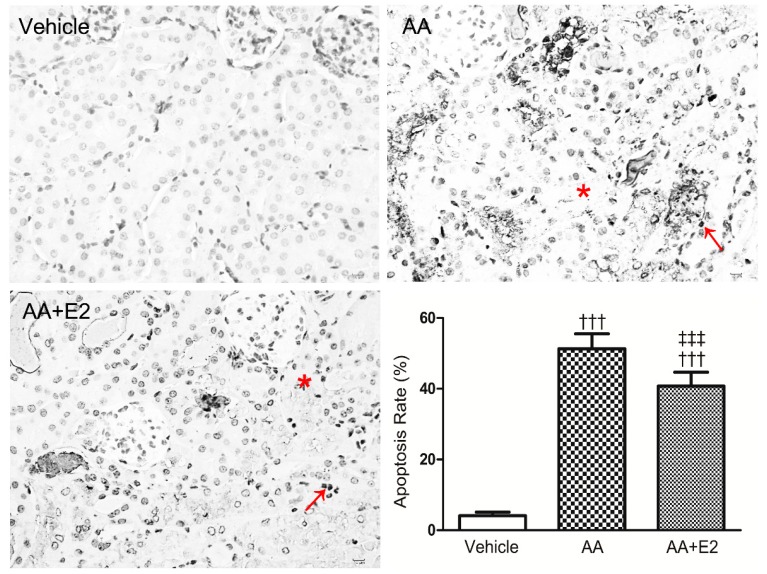
AA-induced RTEC apoptosis (showed by TUNEL-stained sections and quantitative analysis of TUNEL^+^ apoptotic cells) in male mice at day 5 were alleviated by treatment with E2. Note that there were many TUNEL^+^ apoptotic cells (arrow) in the area with cortical tubular necrosis (asterisk) in a male AAN mouse with or without E2 treatment. Data were expressed as means ± SD for groups of 6 mice. ^†††^ *p* < 0.001 vs. vehicle group; ^‡‡‡^ *p* < 0.001 vs. AA group. Magnification, ×200.

The TUNEL assay showed that the number of TUNEL^+^ apoptotic RTECs in the E2-treated group (40.70% ± 3.99%) was less than that of AA group (51.32% ± 4.20%) (*p* < 0.001) (Figure 5). These results highlight that female hormone E2 possess protective effects on renal injury and RTEC apoptosis in acute AAN.

### 2.3. E2 Prevented p53-Related Apoptosis in Renal Tissue of Acute AAN Mice

In our previous study, AA was found to induce RTEC apoptosis by the activation of p53 to promote renal injury in acute AAN. Therefore, we further explored the roles of p53 and cleaved-caspase-3 proteins in the apoptosis of E2-protected kidneys in acute male AAN mice. 

**Figure 6:**
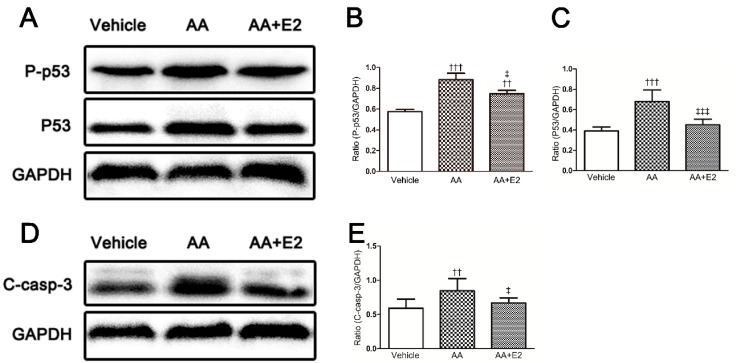
E2 downregulated p53 in renal tissue of acute AAN mice. (**A**) Phosphorylated p53 (P-p53) and p53 expression by western blotting; (**B**) Ratio between P-p53 and GAPDH measured from Western blots; (**C**) Ratio between p53 and GAPDH measured from Western blots; (**D**) Cleaved caspase-3 (C-casp-3) expression by Western blotting; (**E**) Ratio between C-casp-3 and GAPDH measured from Western blots. Data were expressed as mean ± SD for groups of 6 mice. ^††^ *p* < 0.01, ^†††^ *p* < 0.001 vs. vehicle group; ^‡^ *p* < 0.05, ^‡‡^ *p* < 0.01, ^‡‡‡^ *p* < 0.001 vs. AA group.

The data from Western blot analysis indicates that the expression of phosphorylated (Ser 15) p53 (P-p53) and p53 and were significantly increased by AA. The female hormone E2 effectively reduced the overexpression of AA-induced P-p53 and p53 proteins (Figure 6A–C). Furthermore, caspase-3 as an important biomarker of apoptosis, was activated by AA injection, which was indicated by an upregulated expression of cleaved-caspase-3. Significantly, E2 reduced the expression of cleaved-caspase-3 in the E2-treated group compared with that of AA group (*p* < 0.05, Figure 6D,E).

### 2.4. E2 Reduced AA-Induced Apoptosis in HK-2 Cells

**Figure 7:**
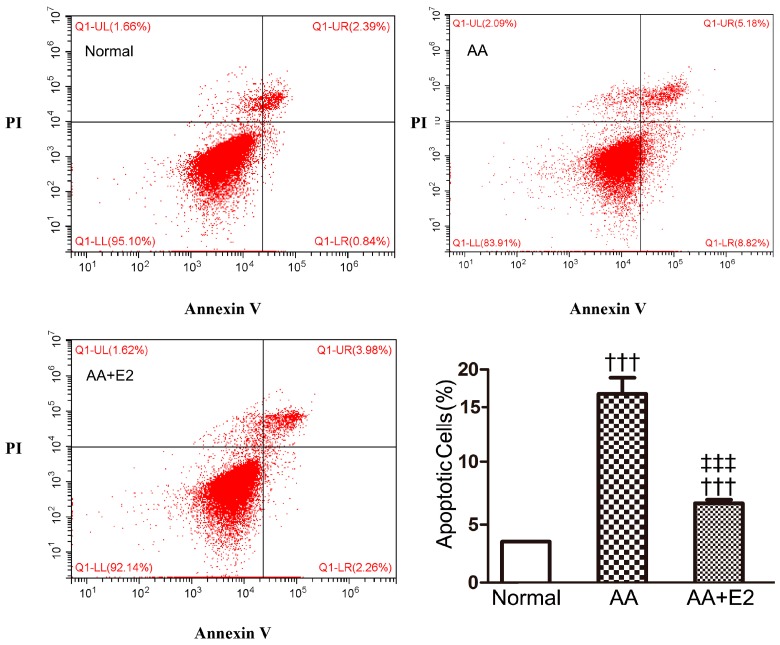
AA-induced HK-2 cells apoptosis, as detected by Annexin V-FITC/PI staining flow cytometry analysis, were blocked by treatment with E2 at 24 h. Annexin V-FITC/PI staining flow cytometry analysis and quantitative analysis of apoptotic cells. The percentage of apoptotic cells was identified by Annexin V^+^. Data were expressed as means ± SD for three independent experiments. ^†††^ *p* < 0.001 vs. normal group; ^‡‡‡^ *p* < 0.001 vs. AA group.

The apoptosis of RTECs characterized the acute phase of AAN. Human RTECs HK-2 cells were used to investigate whether E2 protected AA-induced cell apoptosis in vitro. As shown in Figure 7, AA at 10 μg/mL for 24 h significantly induced apoptosis in HK-2 cells compared with control (15.4% ± 1.28% vs. 3.2% ± 0.01%, *p* < 0.001). Pretreatment with 2 μg/mL of E2 effectively lowered the number of AA-induced apoptotic cells (6.3% ± 0.29%, *p* < 0.001). The in vitro anti-apoptotic result of female hormone E2 and that of male AAN mice access to E2 pretreatment were in the same tendency.

### 2.5. E2 Prevented p53-Dependent Apoptosis in HK-2 Cells

**Figure 8:**
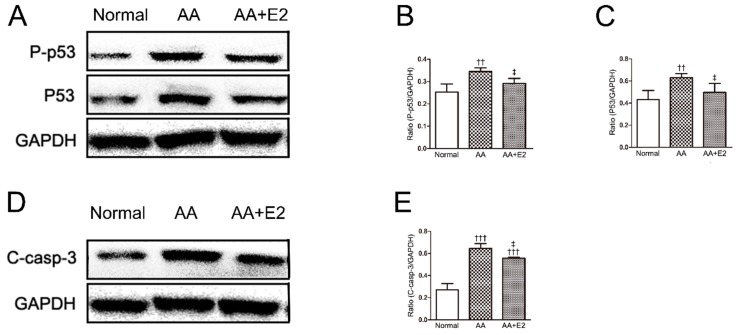
E2 downregulated p53 in HK-2 cells. (**A**) Phosphorylated p53 (P-p53) and p53 expression by western blotting; (**B**) Ratio between P-p53 and GAPDH measured from Western blots; (**C**) Ratio between p53 and GAPDH measured from Western blots; (**D**) Cleaved caspase-3 (C-casp-3) expression by Western blotting; (**E**) Ratio between C-casp-3 and GAPDH measured from Western blots. Data were expressed as mean ± SD for three independent experiments. ^††^ *p* < 0.01, ^†††^ *p* < 0.001 vs. normal group; ^‡^ *p* < 0.05 vs. AA group.

Accordingly, we also investigated in vitro roles of p53 in E2-treated HK-2 cells. Our results showed that AA significantly upregulated the expression of P-p53 and p53 proteins in HK-2 cells, whereas E2 reduced the levels of AA-induced P-p53 and p53 proteins (Figure 8A–C). As for cleaved-caspase-3 protein, the levels of the AA group were increased compared with that of control group, and E2 reduced the expression in the E2-treated AA group (*p* < 0.05, Figure 8D,E). These in vitro results also indicated that pretreatment of female hormone E2 attenuated RTEC apoptosis by the regulation of p53 proteins.

## 3. Discussion

Aristolochic acid nephropathy (AAN) is a progressive kidney disease mainly caused by aristolochic acid in some Chinese herbs, which has been found to lead to ESRD [1,5,6,7]. Excessive death of RTECs characterizes the acute phase of AAN [4,8]. Therapies for AAN are limited, such as steroids and ARBs/ACEIs. In the meantime, an increasing number of studies focus on the role of sex differences in susceptibility to renal diseases and have gradually recognized the renal-protective roles of female hormones [10,11,16,17,18,19,22,23]. However, studies of sex differences and female hormones involved in acute kidney injury are still few.

Sex differences in AA-induced tubulointerstitial fibrosis have been previously reported, and males showed faster progression to renal failure than did females [4]. In our study, we established acute AAN models on the basis of C57BL/6 mice by intraperitoneal injection of AA at 10 mg/kg/day for 4 days. The SCr level of AA-injected male mice saw a 1.5-fold increase compared with saline mice, and the proportion of injured cortical tubule was up to 15%, whereas only a slight increase in SCr without obvious renal tubule injuries was found in females treated by AA. These results suggest that the tolerance to AA in female mice is higher than that in male mice, which is the same conclusion as those of other observations in acute AAN patients ([14,15] and our unpublished data). The possible mechanisms responsible for gender differences in acute AAN might be due to renal-protective effects of estrogen, renal-damage effects of androgen on kidney diseases, or both. It has been reported that E2, with or without orchiectomy treatment, exhibit comparable functions to ameliorate diabetic renal injury [18]. However, our study mainly focused on the protective effects of E2 on renal injury in male mice of acute AAN.

Female hormone E2 supplement has been reported to reduce the level of SCr and proteinuria in a rat remnant kidney model of chronic renal failure [11]. Pretreatment with E2 can attenuate renal dysfunctions in ischemia-reperfusion injury, characterized by the reduction in hemorrhage and tubular necrosis in female mice [17]. E2 administration has a potential effect to improve renal ischemia tolerance in neutered, but not hormonally intact, males [24]. It has been demonstrated that E2 treatment after resuscitation from cardiac arrest can attenuate acute renal injury in young male mice [12]. However, few studies have investigated the effect of E2 in acute renal injury induced by AA of male mice. In this present study, the administration of E2 at a dosage of 2 mg/kg/day effectively improved renal dysfunction and histological damage in acute AAN, indicated by reduced SCr levels and the proportion of injured tubules, respectively. We also found that a low dosage of E2 (80 μg/kg/day) could not reduce SCr level or inhibit injured tubules in acute AAN (data not shown). Akari et al. demonstrated that the E2 level and androgen-to-E2 ratio are pivotal factors in blocking the progression of diabetic renal injury and promoting recovery from diabetic renal injury of mouse models [18]. Thus, renal protection of 17β-estradiol on acute AAN was dose-dependent.

Apoptosis is a main pathological mechanism leading to RTEC deletion and tubular atrophy in AAN [4,8,9,25,26,27]. AA can induce RTEC apoptosis in a dose- and time-dependent manner [8,9,26]. Our study also confirmed via a TUNEL assay that RTEC apoptosis plays dominant roles in AA-induced renal injury, which is consistent with these reported studies concerning renal tubular injury and RTEC apoptosis. In our previous study, we found that the addition of AA at a higher dosage (>10 μg/mL) for 24 h caused significant cytotoxicity in the RTECs of rats [8]. In this study, AA at a concentration of 10 μg/mL for 24 h led to significant apoptosis in cultured HK-2 cells. We also found that the rate of apoptotic RTECs was significantly lowered in the E2-treated AA group compared with the AA group, both in vivo and in vitro. Its beneficial effects seemed partial in terms of the reduction of tubular apoptosis. One of the reasons might be that the tubular cells apoptosis induced by AA is also related to other mechanisms, such as mitochondrial permeability transition [28].

Although E2 protects from RTEC apoptosis, the underlying mechanism that E2 inhibited apoptosis remains unclear. It has been found that RTEC apoptosis induced by AA is mediated by the p53 pathway [8]. AA can produce DNA adducts in RTECs, and DNA damage can activate p53 via posttranslational modifications [29,30,31]. The genotoxic stress of AA-DNA adducts in tubular cells may represent a mechanism by which AA activates p53 [8]. Our data shows that AA increases the expression and phosphorylation of p53 protein, both in vivo and in vitro, highlighting that p53 signaling pathway is involved in AA-induced RTEC apoptosis. The present data are consistent with previous reports [8]. It has been demonstrated that E2 can protect neurons from the amyloid-beta peptide-induced apoptosis by decreasing the p53 protein in an experimental model of Alzheimer’s disease [32]. In breast cancer cells, it has been reported that E2 is protective against p53-mediated apoptosis [33]. The underlying mechanisms by which E2 inhibits the p53 pathway may be related to the expression of genes [32,33]. In our study, E2 significantly reversed the alteration of expression of P-p53 and p53 proteins. This indicates that E2 inhibited the p53 pathway to reduce renal tubular injury of acute AAN. 

## 4. Materials and Methods

### 4.1. Animal Model of Acute AAN

C57BL/6 mice (12-weeks-old; 25–30 g) were purchased from the Animal Laboratory Center of Sichuan University (Chengdu, China). Acute AAN models (*n* = 6) were generated by the intraperitoneal injection (i.p.) of AA (AAI sodium salt; Sigma Aldrich, Shanghai, China) at a dosage of 10 mg/kg/day in 200 μL of saline for 4 days. The control mice were treated with saline at 200 μL/day for 4 days. Mice were randomized to E2 or saline treatment and subsequently underwent experimental procedures. To investigate the renal-protective effect of E2 on renal injury in acute AAN, 17β-estradiol (Sigma Aldrich, China) dissolved in DMSO (Sigma Aldrich, China) at a dosage of 2 mg/kg/day from day 1 to 4 was injected 30 min before the injection of AA (10 mg/kg/day) in male mice. The AAN male mice received the same amount of AA in saline-containing DMSO. The same volume of saline-containing DMSO was injected in the vehicle-treated mice. Experimental animals were randomly divided into three groups and subsequently underwent experimental procedures. Groups of six mice were sacrificed on day 5 after treatment with AA. Terminal blood samples and kidney tissues were collected for further investigations. Animal care methods and experimental protocols were approved by Animal Care and Use Ethics Committee of Committee of Sichuan University in China (IACUC number: 20100318).

### 4.2. Measurement of Renal Function

The levels of serum creatinine (SCr) were used to evaluate renal function. Blood samples were obtained from the heart under anesthesia. The SCr was measured by the method of HPLC-MS/MS.

### 4.3. Histological Analysis

Formalin-fixed, paraffin-embedded kidney sections (4 μm) were stained with periodic acid-Schiff (PAS). Quantification of tubular death in the cortical area on PAS-stained sections using the Image J program (National Institutes of Health) was undertaken as previously described [8]. The cortex with tubular death was identified and outlined. Ten fields (×10) of cortical tissues were counted, and the percentage of death area in the examined cortical area was measured. Data were expressed as a percentage of death area in the cortical area examined.

### 4.4. TUNEL Staining for Apoptosis

TUNEL staining was performed on paraffin-embedded kidney sections using an in situ cell death detection kit (Roche Applied Science) according to the manufacturer’s instructions. The number of TUNEL^+^ cells in the cortical area was counted in five random fields of view on each slide under high-power fields (×400) and expressed as the ratio of the number of TUNEL^+^ cells to that of total cells.

### 4.5. Cell Culture

Human renal proximal tubule cell line (HK-2 cell) was a gift from Prof. Xueqing, Yu (The First Affiliated Hospital, Sun Yat-sen University). HK-2 cells were grown in phenol red-free Dulbecco’s modified Eagle’s medium DMEM/F12 (Hyclone, Beijing, China), containing 10% fetal bovine serum (FBS) at 37 °C in a humidified 5% CO_2_ condition. AA at a dosage of 10 μg/mL was added to the culture for 24 h to induce apoptosis damage. E2 (2 μg/mL) was added 30 min before treatment of AA. Three independent experiments were performed in the study.

### 4.6. Annexin V-FITC/Propidium Iodide (PI) Staining

AA induced HK-2 cells apoptosis was measured by flow cytometer (Beckman Cytoflex, Beckman Coulter Australia Pty Ltd., Lane Cove, NSW, Australia) using the FITC Annexin V Apoptosis Detection Kit I (BD Biosciences, Indianapolis, IN, USA) according to the manufacturer’s instructions. Briefly, HK-2 cells were harvested after various experimental treatments via centrifugation at 1200 rpm for 3 min and washed twice by PBS. HK-2 cells were resuspended in a 300 μL binding buffer, stained with annexin V-FITC solution (3 μL) for 10 min and then stained with a PI (3 μL) solution for 5 min in the dark. Cells (20,000 cells for each sample) were analyzed by flow cytometer. The percentage of early apoptotic (Annexin V^+^PI^–^ cells) and late apoptotic (Annexin V^+^PI^+^ cells) were calculated in total.

### 4.7. Western Blot Analysis

Proteins were extracted from renal tissues or cultured cells with a RIPA lysis buffer and analyzed via Western blotting. Briefly, after the protein was transferred onto a PVDF membrane (Bio-Rad, Hercules, CA, USA), the membrane was blocked with 5% nonfat milk in Tris-buffered saline (TBS) containing 0.1% Tween-20 (TBS/T) for 1 h. The membranes were incubated with primary antibodies against phosphor-p53 (Ser 15) and P53 (Novus Biologicals), cleaved-caspase-3 (Cell Signaling Technology) and GAPDH (Origene) overnight at 4 °C. The membranes were washed three times with 10 mL of TBS/T and then incubated with secondary antibodies (R&D Systems) for 1 h at room temperature. After the membranes were washed three times with TBS/T, the proteins on the membrane were visualized with an enhanced chemiluminescence reagent (Millipore Corporation, Boston, MA, USA). The signals were detected with an Odyssey Infrared Imaging System (Bio-Rad, ChemiDoc MP, mANUSC, Bio-Rad Laboratories Inc., Hercules, CA, USA) and quantified with the Image J program (National Institutes of Health). The ratio for the protein examined was normalized against GAPDH. 

### 4.8. Statistical Analysis

All data were expressed as means ± standard deviation (SD). All results were analyzed using SPSS 16.0 software (SPSS Inc., Chicago, IL, USA). Statistical analyses were performed using a one-way analysis of variance (ANOVA) followed by the Student–Newman–Keuls (SNK) test. Differences between groups were considered significant when *p* < 0.05.

## 5. Conclusions

In summary, this study indicated the tolerance to acute AAN was higher in female mice than that in male mice. Importantly, the female hormone E2 possessed a renal protective effect on male mice with acute AAN, which might consist in inhibiting the p53 signaling pathway to reduce RTEC apoptosis. This might provide a new therapy target for the treatment of acute AAN.
